# Lack of significant association of an insertion/deletion polymorphism in the angiotensin converting enzyme (*ACE*) gene with tropical calcific pancreatitis

**DOI:** 10.1186/1471-230X-6-42

**Published:** 2006-12-12

**Authors:** Seema Bhaskar, DN Reddy, Swapna Mahurkar, GV Rao, Lalji Singh, Giriraj R Chandak

**Affiliations:** 1 Genome Research Group, Centre for Cellular and Molecular Biology, Uppal Road, Hyderabad 500 007, India; 2 Asian Institute of Gastroenterology, Punjagutta, Hyderabad 500 082, India

## Abstract

**Background:**

The genetic basis of tropical calcific pancreatitis (TCP) is different and is explained by mutations in the pancreatic secretory trypsin inhibitor (*SPINK1*) gene. However, mutated *SPINK1 *does not account for the disease in all the patients, neither does it explain the phenotypic heterogeneity between TCP and fibro-calculous pancreatic diabetes (FCPD). Recent studies suggest a crucial role for pancreatic renin-angiotensin system during chronic hypoxia in acute pancreatitis and for angiotensin converting enzyme (*ACE*) inhibitors in reducing pancreatic fibrosis in experimental models. We investigated the association of *ACE *gene insertion/deletion (I/D) polymorphism in TCP patients using a case-control approach. Since *SPINK1 *mutations are proposed a modifier role, we also investigated its interaction with the *ACE *gene variant.

**Methods:**

We analyzed the I/D polymorphism in the *ACE *gene (g.11417_11704del287) in 171 subjects comprising 91 TCP and 80 FCPD patients and compared the allelic and genotypic frequency in them with 99 healthy ethnically matched control subjects.

**Results:**

We found 46% and 21% of TCP patients, 56% and 19.6% of FCPD patients and 54.5% and 19.2% of the healthy controls carrying the I/D and D/D genotypes respectively (P>0.05). No significant difference in the clinical picture was observed between patients with and without the del allele at the *ACE *in/del polymorphism in both categories. No association was observed with the presence or absence of N34S *SPINK1 *mutation in these patients.

**Conclusion:**

We conclude that the *ACE *insertion/deletion variant does not show any significant association with the pathogenesis, fibrosis and progression of tropical calcific pancreatitis and the fibro-calculous pancreatic diabetes.

## Background

Chronic Pancreatitis (CP) is a major health care problem worldwide and is associated with varied etiologies. CP is identified clinically by loss of endocrine and/or exocrine functions and pathologically by the presence of chronic inflammation and fibrosis of the pancreas [1,2]. Tropical calcific pancreatitis (TCP) is a form of chronic pancreatitis of unknown etiology, more prevalent in the tropical regions of developing countries such as India whereas fibrocalculous pancreatic diabetes (FCPD) is a form of diabetes secondary to TCP [3]. Both TCP and FCPD are associated with extensive fibrosis involving both intra and inter-lobular regions and not limited to one zone of the pancreas, although the extent of fibrosis varies and is usually more in FCPD [4]. We and several others previously demonstrated an absence of mutations in the cationic trypsinogen gene (*PRSS1*) and a higher frequency of N34S mutation in the pancreatic secretory trypsin inhibitor (encoded by *SPINK1 *gene) in both FCPD and TCP patients without diabetes mellitus suggesting a common genetic basis for them [5,6]. However, other genetic and/or environmental factors may explain the phenotypic variability in FCPD and TCP patients without diabetes [5].

The renin-angiotensin system (RAS) plays a central role in health and disease by maintaining the electrolyte and fluid balance and in turn, the blood pressure [7]. Apart from circulating RAS, several studies suggest the existence of local RAS components in tissues such as brain, heart, kidney, pancreas, adrenals and gonads [8,9]. Tissue specific RAS can act locally as paracrine and/or autocrine factors in meeting specific needs of individual tissues [10] and any functional alterations may be associated with pathophysiology of respective tissue/organ functions [11-13]. An intrinsic renin-angiotensin system (RAS) is present in the pancreas. Angiotensin converting enzyme (ACE), a zinc metallopeptidase is a key enzyme of this system [14]. ACE and RAS activity is known to be enhanced during acute pancreatitis and chronic hypoxia in experimental animals [15,16]. ACE cleaves angiotensin (AT)-I to AT-II, which induces proliferation of hepatic stellate cells (HSCs) and synthesis of extracellular matrix proteins by increasing the expression of transforming growth factor-beta [17,18]. Isolated pancreatic stellate cells are similar to HSCs [19] and known to be involved in pathogenesis of pancreatic fibrosis in both experimental animals and humans [20]. Increased accumulation of extra-cellular matrix is a histological characteristic of chronic pancreatitis that results in pancreatic fibrosis [21]. In addition, ACE inhibitors have recently been demonstrated to attenuate pancreatic inflammation and fibrosis in spontaneously occurring chronic pancreatitis in male Wistar Bonn/Kobori rats [22].

A polymorphism within intron 16 (g.11417_11704del287) of the *ACE *gene is strongly related to the circulating enzyme levels in a dose dependent manner [23]. The DD genotype that is associated with higher levels of circulating ACE than the II and ID genotypes, is known to be significantly more frequent in patients with myocardial infarction or patients with diabetic proteinuria than in controls [24,25]. Thus, *ACE *gene polymorphisms may play a role in the pathogenesis of chronic pancreatitis and several other pancreatic diseases including acute pancreatitis, pancreatic cancer, diabetes mellitus and cystic fibrosis. Although a recent study failed to find any association of *ACE *I/D polymorphism in familial and sporadic chronic pancreatitis, we hypothesized a role for this polymorphism in view of the extensive fibrosis observed in the TCP patients [26]. In this study, we investigated the *ACE *gene I/D polymorphism in TCP patients (without diabetes mellitus), FCPD patients and healthy controls to understand its association with the disease. Since, *SPINK1 *mutations are proposed to play a modifier role, we also investigated whether any association exists with the I/D polymorphism in the *ACE *gene.

## Methods

### Patients and controls

A total of 171 unrelated patients (120 M+51 F) comprising of 91 TCP and 80 FCPD patients were recruited in the study based on the WHO criteria [27]. 99 unrelated ethnically matched individuals with complaints of dyspepsia were recruited as controls after a similar set of investigations excluded the disease in them. These individuals filled the same questionnaire and underwent similar investigations as the patients especially the imaging procedures which included computed tomography, endoscopic ultrasonography, magnetic resonance cholangio-pancreatography and endoscopic retrograde cholangio-pancreatography, wherever indicated [5]. All the patients and healthy controls were explained the purpose of the study and the complications of investigative procedures and peripheral blood samples were collected after they signed the written informed consent. The study was approved by the institutional ethics committee of respective institutes following the Indian Council of Medical Research guidelines for handling human samples.

### Genetic analysis

**Figure 1:**
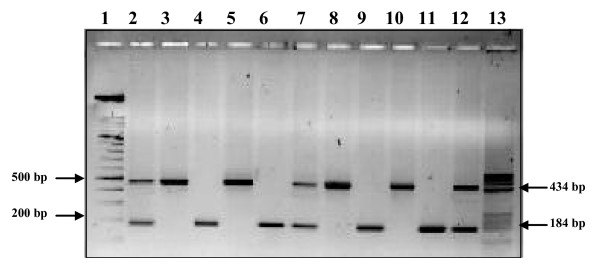
Agarose gel electrophoresis showing genotypes at in/del polymorphism in the *ACE *gene (g.11417_11704del287). Lanes 1 & 13 represent DNA marker XIV (New England Biolabs, USA) and marker V (Boerhinger Manheim, Germany), Lane 2:Control for genotype ID, Lane 3:Control for genotype II, Lane 4:Control for genotype DD. Lanes 5, 8, 10 show II genotype, lanes 7 and 12 are ID genotypes and lanes 6, 9, 11 carry the DD genotype. bp, base-pair

Genomic DNA was isolated from peripheral blood leucocytes following standard protocols [28]. The genotype at I/D variant was determined by PCR amplification of genomic DNA fragment in intron 16 of the *ACE *gene using forward and reverse primers, 5'-CCACTCCCATCCTTTCTCC-3' and 5'-GGCCATCACATTCGTCAGA-3' respectively. The position of the amplified region in the gene covering the polymorphism was 11417–11595 (as per NT_010783). PCR was performed using cycling conditions of initial denaturation at 94°C for 3 min, followed by 35 cycles of denaturation at 94°C for 1 min, annealing at 59°C for 1 min and extension at 72°C for 1 min 30 sec, with a final 5 min extension at 72°C. PCR products of approximate 179 bp (without insertion) and approximate 466 bp (with 287 bp insertion) were detected by electrophoresis on a 2% agarose-gel containing ethidium bromide as shown in figure 1. Genotyping was repeated if any ambiguity was noted in the observations. The primer sequences and PCR conditions for analysis of the promoter region and four exons of the *SPINK1 *gene were selected from Witt et al. [29]. All the exons were sequenced individually on both the strands using internal sequencing primers and Big dye terminator cycle sequencing ready kit and an ABI 3730 Genetic Analyzer (Applied Biosystems) [5]. 10% of randomly selected samples were sequenced to confirm the genotypes.

### Statistical analysis

Allele frequency was calculated by the allele counting method, which was then utilized to determine the genotype frequency. The patients were divided into TCP and FCPD based on the presence or absence of diabetes mellitus and further based on the status of N34S *SPINK1 *mutation and ACE in/del polymorphism. Statistical comparisons were carried out by using Pearson's Chi-squared test, unpaired t-test and odds ratios (OR) along with the 95% confidence interval (CI). Two-tailed P values less than 0.05 were considered statistically significant. Post-hoc power analysis showed the study being adequately powered (84.6%) to accept the null hypothesis that the I/D polymorphism in *ACE *gene is not associated with TCP (α = 0.05, β = -1.426) [30].

## Results and discussion

Tropical calcific pancreatitis is a form of chronic pancreatitis associated with fibrosis and pancreatic calcification. FCPD, a form of diabetes secondary to TCP, is thought to occur due to pancreatic destruction as a result of fibrosis and calcification [3]. We investigated the association of I/D polymorphism in the *ACE *gene with TCP and the phenotype of diabetes in FCPD.

The median age at onset and age at presentation for FCPD patients was 32.6 yrs (95% CI 29.6–35.6) and 40.9 yrs (95% CI 37.8–44.0), which is significantly higher than that of TCP patients without diabetes mellitus [(25.8 yrs, 95% CI 23.5–28.1, P = 0.004) and (33.9 yrs, 95% CI 31.6–36.2, P = 0.001) respectively] (table 1). However, the duration of disease was similar in either category. As suggested earlier, FCPD represents a late stage manifestation of TCP [5,31].

We did not observe any significant difference either in the allele frequency or in the genotype distribution of the I/D polymorphism at *ACE *locus between patients and controls (D allele frequency; 0.46 and 0.47 respectively) and between TCP and FCPD patients (D allele frequency; 0.44 and 0.48 respectively) (table 2). No deviation from Hardy-Weinberg equilibrium was observed (P>0.05). Since frequency of mutated *SPINK1 *is reported to be similar in TCP and FCPD patients [5,6], we investigated whether the I/D variant in *ACE *gene can explain the phenotypic variability between them. We did not observe any association of the *ACE* I/D polymorphism with various phenotypic features including the age of onset and age of presentation, pancreatic calcification or surgeries (table 3). Table 4 summarizes the allele frequency and genotype distribution of I/D polymorphism in patients and the controls based on the presence or absence of N34S *SPINK1 *mutation (irrespective of their zygosity, heterozygous or homozygous genotypes). Of 91 TCP patients, 60 carried the wild type allele while 31 (34%) had at least one copy of the mutant allele at N34S *SPINK1 *variant. Genotypic distribution of the I/D variant was similar in the patient groups with and without N34S mutation in *SPINK1 *gene as well as in TCP and FCPD patient group and the normal individuals. Among TCP patients carrying mutated *SPINK1*, 15 (48%) patients were heterozygous for I/D ACE variant and 5 (16%) carried the DD genotype whereas of 41 patients without N34S *SPINK1 *mutation, 27 (45%) had I/D genotype and 14 (23%) carried DD genotype at the *ACE *locus. Similar observations were made for the group of 80 FCPD patients with and without N34S mutation in the *SPINK1 *gene. This suggests that there is no interaction between the I/D variant in the *ACE *gene and the N34S mutation in the *SPINK1 *gene in either TCP or FCPD patients. It may be interesting to investigate whether other variants in the ACE gene or any specific haplotype may explain the role of ACE in TCP and FCPD.

Angiotensin converting enzyme catalyzes the conversion of angiotensin I into the vasoactive and aldosterone-stimulating peptide angiotensin II [14], which carries out its biological functions by binding to two receptors, AT1R and AT2R [32]. Several studies have suggested the presence of a pancreatic RAS, having physiological effects via a paracrine/autocrine pattern in the exocrine and endocrine pancreas, probably in the regulation of pancreatic microcirculation, ductal anion secretion and islet hormonal secretion [9]. Both circulating and intrinsic pancreatic ACE activity is markedly elevated during acute and chronic inflammation of the pancreas [15,16]. Recent studies have shown that ACE enzyme directs pancreatic fibrogenesis in experimental animals [33] and inhibition of the RAS system with an ACE inhibitor attenuates pancreatic inflammation and fibrosis in an animal model of chronic pancreatitis [22]. Thus, angiotensinogen-II converted endogenously from angiotensinogen-I by circulating ACE may be directly involved in the induction of pancreatic inflammation and development of pancreatic fibrosis in the spontaneously occurring chronic pancreatitis in male Wistar Bonn/Kobori (WBN/Kob) rats [22]. However, our study failed to observe any association between I/D variant and TCP or FCPD patients irrespective of N34S *SPINK1 *mutation status.

Our results are in agreement with the recent study on familial and sporadic chronic pancreatitis patients, which showed that the insertion/deletion polymorphism in the *ACE *gene does not make a significant contribution to the pathogenesis and progression of the disease [26]. Although, recent evidence suggests that TCP is a specific genetic entity [5], there may be certain overlapping features with idiopathic chronic pancreatitis (ICP). In fact, the nomenclature of idiopathic chronic pancreatitis is itself a matter of debate since genetic etiology appears to play an important role in both ICP and TCP in India [34]. Thus, *ACE *I/D polymorphism may not contribute to the pathogenesis of TCP despite the fact that the RAS system specifically affects the pancreas, an indication that local effects are more important than the systemic ones in pancreatic diseases. In the absence of intra-pancreatic ACE levels in these patients, there may exist a possibility that in contrast to other tissues such as heart and renal tissue, DD genotype does not influence the *ACE *gene expression and consequently its levels in the pancreas.

## Conclusion

We conclude that the insertion/deletion polymorphism at the *ACE *locus does not play any role in the causation and progression of TCP. Further studies focusing on other environmental and/or genetic factors such as mutations in the *CFTR *gene may explain the phenotypic variability between TCP and FCPD patients.

## Abbreviations

*ACE *Angiotensin converting enzyme

AT Angiotensin

CP Chronic pancreatitis

CI Confidence interval

FCPD Fibrocalculous pancreatic diabetes

HSCs Hepatic stellate cells

ICP Idiopathic chronic pancreatitis

OR Odds ratio

*PRSS1 *Protease, serine, 1 (trypsin 1)

RAS Renin-angiotensin system

TCP Tropical calcific pancreatitis

*SPINK1 *Serine protease inhibitor, Kazal type I

WHO World Health Organization

## Competing interests

The author(s) declare that they have no competing interests.

## Authors' contributions

SB carried out the molecular genetic studies including sequencing analysis, DNR and GVR designed studies of the phenotypes, identified and diagnosed the patients, SM performed the statistical analysis, GRC conceptualized, designed the study and drafted the manuscript. All authors read and approved the final manuscript.

## Pre-publication history

The pre-publication history for this paper can be accessed here:



## Figures and Tables

**Table 1:** Characteristics of the study population

	**TCP**	**FCPD**	**Total**
**n**	91	80	171
**Sex (Male/Female)**	61 M/30 F	59 M/21 F	120 M/51 F
**Age at onset (yrs)**	25.8 (23.5–28.1)	32.6 (29.6–35.6)	28.0 (26.0–30.0)
**Age at presentation (yrs)**	33.9 (31.6–36.2)	40.9 (37.8–44.0)	36.4 (34.4–38.4)
**Duration of symptoms (yrs)**	8.8 (7.5–10.1)	10.0 (8.4–11.6)	9.3 (8.3–10.3)
**Age at onset of diabetes (yrs)**	NA	33.8 (31.0–36.6)	NA
**Duration of diabetes (yrs)**	NA	7.2 (5.8–8.6)	NA

n, number of patients; yrs, years; TCP, tropical calcific pancreatitis; FCPD, fibro-calculous pancreatic diabetes

NA, not applicable; Figures are quoted as median, range 95% CI

**Table 2:** Allele frequency and genotype distribution of I/D polymorphism at ACE locus in TCP patients & control subjects

	**Patients **			**Controls**	**P Value**		
		
	**TCP**	**FCPD**	**Total**		**TCP vs. FCPD**	**TCP vs. controls**	**FCPD vs. controls**
**n**	91	80	171	99	-	-	-
**Allele frequency**							
**I**	0.56	0.52	0.54	0.53	0.57	0.67	0.89
**D**	0.44	0.48	0.46	0.47			
**Genotype frequency based on the presence or absence of the mutant allele**							
**II**	0.33 (30)	0.24 (19)	0.29 (49)	0.26 (26)	0.16	0.28	0.74
**ID + DD**	0.67 (61)	0.76 (61)	0.71 (122)	0.74 (73)			

n, number of individuals; TCP, tropical calcific pancreatitis; FCPD, fibro-calculous pancreatic diabetes; ACE, angiotensin converting enzyme; I/D, insertion/deletion polymorphism at ACE locus figures in parentheses indicate number of individuals; P values are represented on comparison of TCP with FCPD, TCP with controls and FCPD with controls.

**Table 3:** Clinical features of TCP and FCPD patients grouped according to their genotype at in/del polymorphism at ACE gene

	**TCP**			**FCPD**			**Total**		
			
	**II**	**ID/DD**	**P value**	**II**	**ID/DD**	**P value**	**II**	**ID/DD**	**P value**
**n**	30	61	-	19	61	-	49	122	-
**Sex (Male/Female)**	22/8	39/22	-	17/2	42/19	-	39/10	81/41	-
**Age at onset (yrs)**	25.7 ± 11.1	24.8 ± 11.8	0.72	34.4 ± 15.1	31.2 ± 12.9	0.37	29.0 ± 13.3	28.0 ± 12.7	0.64
**Age at presentation (yrs)**	36.2 ± 11.8	32.7 ± 10.9	0.16	40.3 ± 17.0	38.9 ± 13.9	0.72	37.8 ± 14.0	35.8 ± 12.9	0.37
**Age at onset of diabetes (yrs)**	NA	NA	NA	34.2 ± 13.9	33.7 ± 11.44	0.88	34.2 ± 13.9	33.7 ± 11.4	0.81
**Pancreatic surgery**	11 (36.67%)	24 (40%)	0.76	2 (10.53%)	4 (6.56%)	0.57	13 (26.53%)	28 (23.14%)	0.64
**Calcification**	30 (100%)	61 (100%)	1	19 (100)	61 (100)	1	49 (100)	122 (100)	1
**Pseudocyst**	2 (6.67%)	4 (6.6%)	1	0 (0%)	1 (1.64)	1	2 (4.08%)	5 (4.13%)	1

n, number of individuals; TCP, tropical calcific pancreatitis; FCPD, fibro-calculous pancreatic diabetes; ACE, angiotensin converting enzyme

I/D, insertion/deletion polymorphism at ACE locus; NA, not applicable; figures in parentheses indicate percentage; figures are quoted as mean ± SD

P values are represented on comparison of II vs ID+DD genotypes in TCP, FCPD and total patients

**Table 4:** Allele frequency and genotype distribution of I/D polymorphism at ACE locus in TCP patients and controls based on their N34S SPINK1 status

	**Patients**								**Controls**		
		
	**TCP**		**P Value**	**FCPD**		**P Value**	**Total**		**P Value**		
						
**N34S SPINK1 mutation status**	Mutant*	**Wild**		**Mutant**^#^	**Wild**		**Mutant**	**Wild**		**Mutant**^$^	**Wild**
	
**N**	31	60		21	59		52	119		3	96
**Allele frequency**											
**I**	0.60	0.54	0.39	0.55	0.51	0.57	0.58	0.53	0.48	0.67	0.53
**D**	0.40	0.46		0.45	0.49		0.42	0.47		0.33	0.47
**Genotype frequency based on the presence or absence of the mutant allele**											
**II**	0.36 (11)	0.32 (19)	0.55	0.29 (06)	0.22 (13)	0.26	0.33 (17)	0.27 (32)	0.35	0.33 (01)	0.26 (25)
**ID + DD**	0.64 (20)	0.68 (41)		0.71 (15)	0.78 (46)		0.67 (35)	0.73 (87)		0.67 (02)	0.74 (71)

n, number of individuals; TCP, tropical calcific pancreatitis; FCPD, fibro-calculous pancreatic diabetes; ACE, angiotensin converting enzyme; I/D, insertion/deletion polymorphism at ACE locus figures in parentheses indicate number of individuals; P value is represented on comparison of N34S SPINK1 positive vs. N34S negative TCP, FCPD and total patients.

*, includes 9 N34S SPINK1 homozygotes and 22 heterozygotes; # includes 4 N34S SPINK1 homozygotes and 17 heterozygotes, $, includes 3 N34S SPINK1 heterozygotes
